# Detecting and Managing Childhood Onset Hypertension in Africa: A Call to Action

**DOI:** 10.1007/s11906-023-01247-3

**Published:** 2023-06-15

**Authors:** A. Craig, Y. Breet, L. F. Gafane-Matemane, S. A. Norris, R. Kruger

**Affiliations:** 1 SAMRC/Wits Developmental Pathways for Health Research Unit, Faculty of Health Sciences, University of the Witwatersrand, Johannesburg, South Africa; 2 Hypertension in Africa Research Team (HART), North-West University, Private Bag X6001, Potchefstroom, 2520 South Africa; 3 MRC Research Unit for Hypertension and Cardiovascular Disease, North-West University, Potchefstroom, South Africa; 4 School of Human Development and Health, University of Southampton, Southampton, UK

**Keywords:** Adolescents, Africa, Cardiovascular disease, Children, Prevention, Primary hypertension, High blood pressure

## Abstract

**Purpose of Review:**

To review recent evidence on childhood hypertension across Africa, identifying knowledge gaps, challenges and priorities, and highlight clinical perspectives in managing primary hypertension.

**Recent Findings:**

Only 15 of the 54 African countries reported on absolute blood pressure (BP) measures, elevated BP, pre- and/or hypertension. The reported hypertension prevalence ranged between 0.0 and 38.9%, while elevated BP and/or pre-hypertnesion ranged from 2.7 to 50.5%. Childhood BP nomograms are lacking across Africa and the rates of hypertension were based on guidelines developed in countries with the lowest to no number of children from African ancestry. The recent studies across Africa also showed little to no detail when reporting BP specific methodology. No recent data informing the use or effectiveness of antihypertensive agents in children and adolesents are available.

**Summary:**

Childhood hypertension is on the rise, while data from Africa remains vastly under-represented. Collaborative research, resources, and policies need to be strengthened in addressing the growing public health concern of childhood onset hypertension on this continent.

## Introduction

Paediatric hypertension—defined by age, sex and height-specific systolic and/or diastolic blood pressure (BP) nomograms—has its roots in early childhood and adolescence [1] as reported in several longitudinal cohorts [2–5]. Hypertension is a public health concern, particularly in Africa where the prevalence is steadily increasing among children and adolescents [6]. With a shift towards elevated BP during childhood as a key driver of adult hypertension, the importance of early elevated BP detection, prevention, and intervention strategies is critical [7].

Hypertension can be stratified by its aetiology into:

1. Primary or essential hypertension that remains a condition of multifactorial origin as a result of behavioural, environmental or genetic causes or the interaction of both.
2. Secondary hypertension that has multiple aetiologies, including renal, vascular, and endocrine causes.

Although secondary hypertension is more common in children than in adults, primary hypertension in the paediatric population is on the rise [6]. Children and adolescents with primary hypertension present with a typical clinical phenotype similar to the abnormalities observed in hypertensive adults. Behavioural, environmental, biological and/or atypical genetic factors mediate the tendency to develop hypertension [8, 9]. While studies have shown a behaviour-environment association with childhood BP, there remains a lack of large-scale genome-wide association studies specific to the African region, and more so, in children and adolescents. The few genetic studies in children and adolescents reported consistent findings for multiple single nucleotide polymorphisms (as a genetic risk score) to associate with BP levels in children [3, 10] and to predict hypertension in adulthood [11]. Although these landmark studies were limited to the Young Finns Study [12], the Avon Longitudinal Study of Parents and Children [13], and the Western Australian Pregnancy Cohort [14], they do provide important information going forward for new studies to determine similarities or differences in genetic variants in other parts of the world. However, in Africa, 50% of African countries are low-income, and 18 African countries are among the world’s poorest [15]. Consequently, the prevalence and concomitant research of childhood hypertension in Africa is limited as many countries are not monitoring and reporting BP in children given other more pressing demands.

Reliable estimates of childhood hypertension serve as the basis for prevention, treatment, intervention and evidence-based health resource allocation and policy making. However, in Africa, childhood hypertension has rarely been synthesised. Therefore, aside from reporting the recent prevalence of childhood hypertension across Africa, this review will also discuss the impact of risk factors and/or interventions associated with childhood BP; current challenges and priorities; and the clinical perspectives surrounding childhood hypertension across the continent.

## Childhood Hypertension in Africa

### The Prevalence of Childhood Hypertension in Africa

**Fig. 1:**
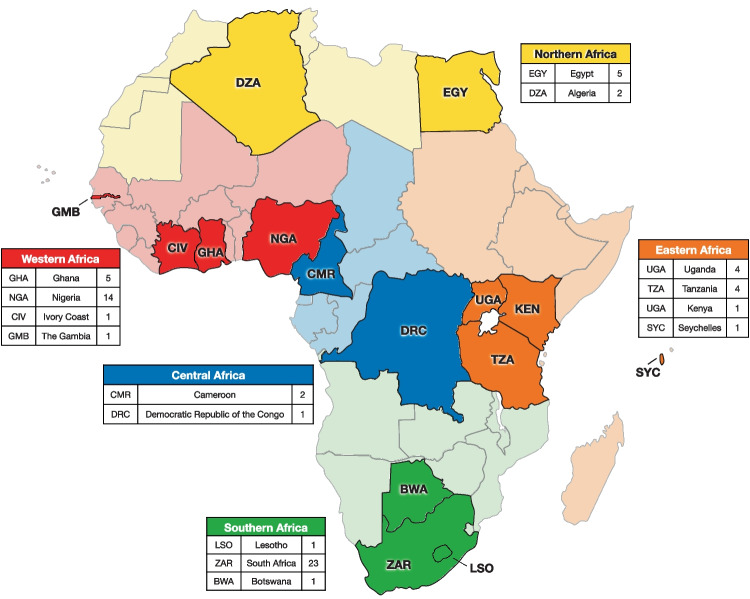
Schematic illustration of the number of studies highlighting the prevalence of hypertension, pre-hypertension and elevated blood pressure across the African continent within the last 5 years

Of recent (2018–2022) studies that included a broad range of randomised control trials (RCTs), interventions, cohort, case, longitudinal and cross-sectional studies, only 66 studies (Table 1) from 15 countries across Africa reported on BP in African children and adolescent cohorts, either absolute BP measures, elevated BP, pre- and/or hypertension. Considering that Africa is the second largest and second most populous continent, after Asia, childhood hypertension in this setting is underreported (Fig. 1). Majority of the studies were those conducted in the Southern most part of Africa (37.9%), with South Africa contributing to 23 of the 25 studies reported in this region. Although not all studies reported a hypertension prevalence, we found that from the 53 studies that did, the reported childhood hypertension prevalence on the African continent between the years 2018 to 2022 ranged extensively from 0.0% in an Egyptian adolescent study that included *n* = 77, 12–18-year olds [16] to 38.9% in 2 studies namely a case control study that included *n* = 72 Egyptian children and adolescents (3–14 years) [17] and that of a RCT on *n* = 1119 Ugandian adolescents (10–11 years) [18].

**Table 1:** Studies highlighting the prevalence of hypertension, pre-hypertension and elevated blood pressure across the African continent within the last 5 years

Author	Data collected	Study design	Country	Setting	*n*	Age range (years)	BP device	#BPs/interval	Guideline	HTN %	EBP/pre-HTN %
El-Koofy et al. [17]	2016	Case control	Egypt		72	3–14			4th Report [82]	38.9	
Elseifi et al. [20]	2017–2018	Intervention	Egypt		224	12–14	Manual	2/2 min ‡	AAP [77]		SBP: 3.6. DBP: 2.7
Hassan et al. [16]	2013–2017	Cohort prospective	Egypt		77	12–18	Manual	2	4th Report [82]	0.0	40.3
Sherif et al. [97]	2016–2017	Cross-sectional	Egypt	Urban	110	4–18					SBP: 9.0; DBP: 10.0
Benmohammed et al. [98]	2007	Cross-sectional	Algeria	Urban	1100	12–18	Manual	3/5 min	4th Report [82]	12.4	13.0
Redjala et al. [50]	2014–2015	Cross-sectional	Algeria	Suburban	3562	6–18	Automated (Omron 705 IT)	2	4th Report [82]	13.6	10.0
Hassan et al. [21]	2013–2016	Cross-sectional	Egypt		200	12–18			4th Report [82]		50.5
Lule et al. [18]	2014–2016	RCT	Uganda	Rural	1119	10–11	Automated (Omron M6, HEM-700)	3/5 min ‡	4th Report [82]	38.9	33.3
Sungwa et al. [29]		Cross-sectional	Tanzania	Urban	742	6–16	Automated (CONTEC 08A)	3/5–10 min	AAP [77]	8.5	9.6
Kansiime et al. [40]		Cross-sectional	Uganda	Rural	1913	6–12		‡			7.8
Katamba et al. [99]	2018	Cross-sectional	Uganda	Peri-urban	616	12–19	Automated (Scian SP-582)	3/5 min ‡	4th Report [82]	3.1	7.1
Gewa et al. [39]		Cross-sectional	Kenya	Urban	390	10–12	Manual			14.0	20.0
Nsanya et al. [100]	2015	Cross-sectional	Tanzania/ Uganda	Urban	827	12–17	Automated (Omron M6)	3/2 min	4th Report [82]	12-14yrs: 15; 15-17yrs: 15	12-14yrs: 16; 15-17yrs: 23
Nyangasa et al. [101]	2013	Cross-sectional	Tanzania/ Zanzibar	Rural	165	5–18			4th Report [82]	9.8	15.8
Leyvraz et al. [102]	1998–2006	Cross-sectional	Seychelles		4519	5–16	Automated (Omron M5)	2/1 min ‡	4th Report [82]	10.2	5.5yrs: 10; 9.2yrs: 10; 12.5yrs 7; 15.6yrs: 9
Muhihi et al. [103]		Cross-sectional	Tanzania	Urban	446	6–17	Automated (Omron Digital HEM-907)	3/5–10 min	4th Report [82]	10.8	4.9
Nakiriba et al. [104]		Cross-sectional	Uganda	Peri-urban	688	12–19	Automated (Welch-Allyn)	3	SBP/DBP > 95th percentile	11.6	
Ndongala et al. [105]	2020–2021	Retrospective	Lesotho	Rural	352	< 18				9.0	
Jourbet et al. [41]	2018	RCT	South Africa	Rural	1009	8–13	Automated (Omron M6 AC)	3/1 min	Neuhauser [90]	18	SBP: 6; DBP: 10
Nqweniso et al. [43]	2015–2016	Cluster RCT	South Africa	Urban	842	8–13	Automated (Omron M6 AC)	2	Neuhauser [89]	13.5	
Masocha et al. [106]	2011–2013	Longitudinal	South Africa		186	14–16	Automated (Omron MIT Elite Plus)	2/5 min ‡	NCEP/ATPIII [107]		10
Boerstra et al. [51]	2019	Cross-sectional	South Africa	Urban	189	3–6	Automated (Dinamap)	3/1 min	AAP [77]	32.3	17.5
Kruger et al. [27••]		Cross-sectional	South Africa	Urban/rural	1062	5–9	Automated (Omron HBP-1100-E)	5/2 min	AAP [77]	22.8	14.1
Letswalo et al. [32]		Cross-sectional	South Africa	Rural	800	13–16	Automated (Omron HBP-1100)	3/2 min	CDC [108]	23.3	15.5
Arnaiz et al. [109]	2019	Cross-sectional	South Africa	Peri-urban	897	8–16	Automated ( Omron M6AC)	2	AAP [77]; Neuhauser [90]; Xi [91], Muller [92]	APP: 28.7; Neuhauser: 29; Xi: 25.6; Muller: 11.3	APP: 9.5; Neuhauser: 7.2; Xi: 11.4; Muller: 6.4
Kochli et al. [110]	2019	Cross-sectional	South Africa	Urban/rural	929	5–9	Automated (Omron HBP-1100-E)	5/2 min	AAP [77]		SBP: 33.4; DBP: 25.6
Ware et al. [111]	2019–2020	Cross-sectional	South Africa	Urban	65	4–9	Automated (SphygmoCor)	3		19.0	8.0
Mokgwathi et al. [37]	2015–2016	Cross-sectional	Botswana	Urban/rural	252	< 18	Automated (BPCB0A–2H)	2/5 min‡	4th Report [82]	13.1	15.5
Gomwe et al. [34]		Cross-sectional	South Africa		876	9–14	Automated (Omron HEM705 CP)	3/5 min	JNC7 [112]	SBP: 5.3; DBP: 2.6	SBP: 18.4; DBP: 14.7
Chungag et al. [113]	2016	Cross-sectional	South Africa	Urban	540	10–14			4th Report [82]	20.7	12.2
Mphekgwana et al. [114]		Cross-sectional	South Africa	Rural	1811	8–17	Automated	3/5 min‡	4th Report [82]	1.3	
Nkwana et al. [115]		Cross-sectional	South Africa	Rural	1665	5–15	Automated	2	JNC7 [112]	14.4	
Houle et al. [116]	2012–2014	Cross-sectional	South Africa	Rural	1536	7–11	Automated (A&D Medical, Model UA-767 Plus 30)	2	4th Report [82]	4.2	
Matjuda et al. [117]		Cross-sectional	South Africa	Urban/rural	306	6–9	Automated (Omron M500, HEM-7321-D)	3/2 min	AAP [77]	10.5	32.3
Matjuda et al. [36]		Cross-sectional	South Africa	Urban/rural	306	6–9	Automated (Omron M500, HEM-7321-D)	3/2 min	AAP [77]		42.3
Sebati et al. [118]		Cross-sectional	South Africa		1665	5–15	Automated	3/5 min	JNC7 [112]	4.3	SBP: 5.3; DBP: 5.6
Raphadu et al. [35]		Cross-sectional	South Africa		218	13–19	Automated (Omron)		4th Report [82]	17.1	27.3
Bhimma et al. [119]	2016–2017		South Africa	Urban	564	8–18			4th Report [82]	13.7	
Meer et al. [120]	1995, 1998, 2002, 2003, 2005, 2008	Cross-sectional	South Africa	Urban	1891	5–18 years	Automated (5yrs: Dinamap 1846SX; 8yrs + : OMRON M6)		4^th^ Report [82]		42
Craig et al. [81••]		Cross-sectional	South Africa	Urban	> 1200	5–17 years	Automated (5yrs: Dinamap 1846SX; 8yrs + : OMRON M6)	3/2 min	AAP [77]; 4^th^ Report [82]; ESH 2016 [78]	AAP: 28.6; 4^th^ Report: 12.5; ESH: 21.6	AAP: 17.3; 4^th^ Report: 26.1; ESH: 13.0
Gerber et al. [121]	2015–2016	Cross-sectional	South Africa	Urban	801	8–13	Automated (Omron, M6 AC)	2	Neuhauser [90]	32.6	
MOmoniyi et al. [45]	2018	Intervention (pilot study)	Ghana		78	9–12	Automated (Omron, HEM-7120-E)				
Oluwayemi and Oyedeji [30]	2010–2020	Cross-sectional	Nigeria		21	1–16			4th Report [82]	19.0	
Uchenwa-Onyenegecha et al. [122]		Cross-sectional	Nigeria		1117	6–16		3		4.4	4.3
Azupogo et al. [123]	2014	Cross-sectional	Ghana	Urban/rural	1727	15–19		3	4th Report [82]	0.2	20.4
Abu et al. [124]	2015	Cross-sectional	Nigeria		420	10–19			4th Report [82]	6.9	8.8
Ukoh et al. [33]	2015–2016	Cross-sectional	Nigeria	Urban	2401	10–19	Automated (OMRON M10-IT)	3/2 min ‡	4th Report [82]	4.6	
Musa et al. [125]	2019	Cross-sectional	Nigeria	Rural	197	11–18	Automated (Omron, HEM-705 CP)	2/2 min	SBP/DBP 130/85	SBP: 5.1; DBP: 12.2	
Akinbodewa et al. [31]		Cross-sectional	Nigeria	Rural	114	3–9	Manual (Accossons)		4th Report [82]	7.0	12.3
Amponsem-Boateng et al. [126]	2018–2019	Cross-sectional	Ghana	Urban	699	15–17	Automated (MOTECH TrueScan) / Manual	1	JNC7 [112]	3.3	3.3
Fossou et al. [42]	2018	Cross-sectional	Ivory Coast	Urban	1251	5–15					26.9
Amponsem-Boateng et al. [38]	2018–2020	Cross-sectional	Ghana	Urban	3165	12–22		3	JNC7 [112]	19.9	26.1
Afaa et al. [28••]		Cross-sectional	Ghana		600	5–14	Automated	3	CDC [108]	2.5	6.0
Ibrahim et al. [127]	2014–2015	Cross-sectional	Nigeria	Urban/rural	1745	6–12	Manual	2/1 min	4th Report [82]	3.0	
Abiodun et al. [128]	2014–2017	Cross-sectional	Nigeria		6980	15–19			AAP [77]	25.3	25.1
Amadi et al. [129]	2017	Cross-sectional	Nigeria		491	6–17	Manual (Riester sphygmomanometer)	3/30 min	JNC7 [112]	9.0	
Adeomi et al. [130]		Cross-sectional	Nigeria		313	10–19	Automated (OMRON 2 digital)	2/10 min	4th Report [82]	6-12yrs: 9.4; 13-17yrs: 6.5	
Schoenbuchner et al. [131]	2012–2015		Gambia	Rural	2773	10–19	Automated (Omron 705-CPII)		4th Report [82]		
Ezeudu et al. [132]	2013–2014	Cross-sectional	Nigeria	Urban	984	10–19	Manual (Accoson)	2/1–2 min	4th Report [82]	5.4	
Omisore et al. [133]	2012	Cross-sectional	Nigeria	Urban/rural	1000	10–16	Manual (Accson)	2	4th Report [82]	4.1	
Iseuzo et al. [134]	2014–2015	Cross-sectional	Nigeria		800	10–18		‡	4th Report [82]	3.1	7.5
Wariri et al. [135]	2019	Cross-sectional	Nigeria	Rural	367	10–18	Automated (Omron HBP-1100-E)	5/2 min	4th Report [82]	5.7	SBP: 33.4; DBP: 25.6
Kaze et al. [49]	2018	Cross-sectional	Cameroon		80	5–15	Automated (OMRON HEM705CP)	3		2.5	
Chelo et al. [136]	2017–2018	Cross-sectional	Cameroon	Urban/rural	822	5–17	Manual (GIMA)	2/30 s‡	AAP [77]	1.6	8.2
Muyumba et al. [22••]	2013–2016		DRC		7523	3–17					
Crouch et al. [23••]	2017–2020	Systematic review/meta-analyses	Africa		53 papers	< 17	-	-	-	7.5	11.4

*n* number of participants, *BP* blood pressure, *HTN* hypertension, *EBP* elevated blood pressure, *RSA* Republic of South Africa, *DRC* Democratic Republic of Congo, *TZ* Tanzania, *UG* Uganda, *SBP* systolic blood pressure, *DBP* diastolic blood pressure, 4^th^ report—The Fourth Report on the Diagnosis, Evaluation, and Treatment of High Blood Pressure in Children and Adolescents [81••], *AAP* American Academy of Pediatrics “Clinical Practice Guideline for Screening and Management of High Blood pressure in Children and Adolescents [76], *JNC7* The Seventh Report of the Joint National Committee on Prevention, Detection, Evaluation, and Treatment of High Blood Pressure [111], ESH 2016:European Society of Hypertension guidelines for the management of high blood pressure in children and adolescents [77], *CDC* Centre for Disease Control and Prevention-National Health and Nutrition Examination Survey [107],* NCEP/ATP III* National Cholesterol Education Program/Adult Treatment Panel III [106]

^‡^Studies that repeated blood pressure on separate occasions

Both aforementioned Egyptian [17] and Ugandian [18] studies that reported an alarmingly high prevalence of childhood hypertension in Africa (38.9%) were suggestive that the level of adiposity or body weight in early life may directly influence BP. El-Koofy et al. showed that babies with a low birth weight are at increased risk of high BP in later life due to rapid early weight gain [17]. Lule et al. showed that hypertension was reported in 70.0% of children with obesity determined via Centre for Disease Control (CDC) anthropometric z-scores [18]. These results are therefore suggestive that the rates of hypertension in childhood are increasing with adverse lifestyle behaviours (i.e. childhood obesity) [19].

Northern Africa, specifically Egypt, reported the lowest and highest prevalence of elevated BP or pre-hypertension across Africa, ranging from 2.7% in an intervention study of *n* = 224 adolescents (12–14 years) [20] to 50.5% in a cross-sectional study of *n* = 200 adolescents (12–18 years) [21]. The intervention study by Elseifi et al. suggested that the students who were pre-hypertensive for systolic and diastolic BP showed infrequent intake of breakfast per week [20], while the cross-sectional study by Hassan et al., showed that girls with overweight/obesity presented with significantly higher prevalence of hypertension (66.7% vs. 40.8%), diabetes (46.7% vs. 31.2%) and low levels of high-density lipoprotein (64% vs. 59.2%) than normal-weight girls [21]. The largest study was a cross-sectional study that was carried out by Muyumba et al. (2018), that included *n* = 7523 children and adolescents (aged 3 to 17 years) in Lubumbashi, Democratic Republic of Congo [22••]. This was the first study of its kind to determine threshold percentiles (50, 90 and 95%) for BP specific to age and height for this unique population demographic [22••].

The only systematic review and meta-analysis between 2018 and 2022 pertaining to childhood hypertension in Africa, was conducted by a group of researchers from South Africa that shed light on childhood hypertension across the continent [23••]. Crouch et al. reported that the prevalence of childhood hypertension in Northern and Eastern Africa, accounted for more than 9.5% in these regions [23••]. Significant differences in the hypertension prevalence was also reported with the Southern and Western African regions trailing close behind (Northern (15.2%), Eastern (9.5%), Southern (7.9%), Western (6.0%), and Central (1.6%)) [23••]. The hypertension prevalence in Africa did not significantly differ when looking at population demographics such as age, sex or areas of urbanicity [23••], and can partially be explained by the lack of geographical data from 18 of the 41 studies. Discrepancies in rural–urban differences are well reported in the African context [24, 25••], but the rise in obesity may supersede the urban-rural divide. Although environmental variations of BP seem difficult to justify, a low socioeconomic status, as seen across many African countries, and the incidence of obesity are among the two most probable contributing factors [26, 27••].

### Blood Pressure Measurement Techniques and Devices Currently in Use Across Africa

BP measurements at a population level can generate important trends to use as an indicator of population health as a whole. Consequently, global guidelines for proper BP measurement have existed for several years. Despite this, a study conducted by Hassan et al., reported a 0.0% childhood hypertension prevalence in Northern Africa but only recorded BP manually and a single measurement [16]. El-Koofy et al., did not disclose or describe any particular BP methods [17] while Lule et al., reported a 38.9% childhood hypertension prevalence after BP was measured on an automated osciillometric device on 3 separate occasions [18] as suggested by known international guidelines. In several research studies, BP measurement methods and thresholds used are not always properly reported, which can impact on results derived from these studies. We found that from the studies we reported in Table 1, the type of BP measurement device, the number of measurements and/or rest intervals between measurements were generally poorly reported. For instance, 18.2% (12/66) of the reported studies did not disclose or describe detailed BP methodology altogether and a further 31.8% (21/66) had missing information regarding either the type of BP measurement device, the number of measurements and/or the resting interval between meaurements. Significant differences have been reported between the type of BP measurement device used (i.e. manual versus automated oscillometric), the number of measurements taken (i.e. single versus multiple/single versus repeated measurement) and the paediatric clinical practice guideline used to stratify participants into their BP statuses. We noted that the majority of studies in the Northern African region still prefer to acquire BP manually. The prevalence of adverse BP across Africa, must be interpreted with caution firstly considering the lack of countries reporting (Fig. 1; Table 1) and secondly considering the array of BP methodology still currently in use across the continent.

### Recent Advances in Childhood Hypertension

Various modifiable risk factors have been linked to higher BP levels and other forms of cardiovascular disease risk in children in Africa (Table 2). These factors have been reported in children and adolescents between the ages of 1–19 years, with the majority of the studies done in Western and Southern African regions. Among the BP associated risk factors, the majority of the findings showed an adverse association between *overweight/obesity* and pre-hypertension and/or hypertension [27••, 28••, 29–40]. The prevalence of overweight/obesity ranged from 2.2 to 33.0% with the highest prevalence being reported in South Africa. The largest study was a national cross-sectional study done by Amponsem-Boateng et al., and included *n* = 3165 students aged between 12 and 22 years [38]. This study aimed to screen for hypertension, risks and knowledge/awareness in second-cycle schools in Ghana. The risk factors for early hypertension that were identified included body mass index (BMI), waist circumference and body weight with a high statistical significance between BMI and pre-hypertension as well as hypertension [38]. In these Ghanaian students, 19.9% were hypertensive and 26.1% were pre-hypertensive which may indicate a likely high prevalence of hypertension in the future adult population [38]. It is noteworthy to mention that the majority of the studies in Africa investigating overweight/obesity in children were cross-sectional in design and that different classifications for overweight/obesity were used throughout (i.e. International Obesity Task Force, World Health Organisation (WHO) and CDC).

**Table 2:** Most recent studies highlighting the prevalence of hypertension risk factors across the African continent

Author	Country	*n*	Age range (years)	Prevalence of risk factor (%)	Comment
**Overweight/obesity**					
Kruger et al. [27••]	South Africa	1062	5–9	20% overweight/obese (BMI z-score > 85th percentile—WHO)	51–60% increased risk of EBP for 1SD increase of sex-specific BMI and WTHR
Afaa et al. [28••]	Ghana	600	5–14	5.9% of children with obesity had EBP (self-reported)	10.5% of participants with EBP had risk factors (family history of HTN; DM; obesity; smoke (self-reported))
Sungwa et al. [29]	Tanzania	742	6–16	15.2% overweight/obese (WHO standards)	30.7% (overweight) and 36.8% (obese) had EBP. Children from urban versus rural areas more likely to have EBP. EBP associated with obesity, overweight, eating fried food, drinking sugar soft drinks and not eating fruits
Oluwayemi et al. [30]	Nigeria	21	1–16	19% overweight/obese (BMI > 95^th^ percentile—CDC)	Children with BMI > 30 had significantly higher rates of HTN
Akinbodewa et al. [31]	Nigeria	114	3–9	6.1% overweight; 6.1% obese (BMI > 85^th^ percentile—WHO)	The most frequently occurring risk factors in the clusters were pre-HTN (25%), low level of high HDL-c (25%), high level of non-HDL-c (25%) and obesity (25%)
Letswalo et al. [32]	South Africa	800	13–16	33% overweight/obese (BMI cutoff—International Obesity Task Force)	61.1% of those with HTN were obese/overweight
Ukoh et al. [33]	Nigeria	2401	10–19	6.8% overweight; 1.3% obese (BMI > 85^th^ percentile—CDC)	3.4% of those with HTN consume junk food, 12.6% consume alcohol and 4.6% had family history of HTN (self-reported)
Gomwe et al. [34]	South Africa	876	9–14	3.7% overweight, 2.2% obese (BMI—WHO)	The proportion of EBP was lower for underweight (18.0%) and normal weight (31.9%) as compared to 43.8% among overweight. An increase in BMI was significantly associated with EBP
Raphadu et al. [35]	South Africa	218	13–19	Overweight: 11.5% obesity: 5.5% (BMI > 30 kg/m^2^—WHO)	7.3% (pre-HTN) and 2.7% (HTN) were overweight/obese. BMI associated with SBP and DBP. BSA associated with SBP and DBP
Matjuda et al. [36]	South Africa	306	6–9	19.3% overweight/obese (BMI > 95^th^ percentile—CDC)	Obesity and HTN associated with renal-CVD risk
Mokgwathi et al. [37]	Botswana	252	< 18	10.3% overweight/ obese (BMI z-score > + 1SD WHO)	HTN, overweight/obesity and alcohol intake (9.1%—self-reported) were common among these adolescents in Botswana
Amponsem-Boateng et al. [38]	Ghana	3165	12–22		Risk factors to early HTN among include age, BMI, wc, height, and weight. A high statistical significance was found between BMI and pre-HTN and HTN
Gewa et al. [39]	Kenya	390	10–12		Overweight (BMI-for-age percentiles) children with EBP was 1.85-fold greater and the proportion of children with HTN was 1.83-fold greater compared with normal weight children. Similar patterns of significant associations were seen among obese children, those with central obesity and those with high total skinfold values
Kansiime et al. [40]	Uganda	1913	6–12		Higher BMI associated with higher BP. Obesity was largely irrelevant in this study
**Physical activity**					
Jourbet et al. [41]	South Africa	1009	8–13	39% physically inactive (self-reported)	Hypertensive children were more likely to be overweight/obese, but only if they did not meet physical activity recommendations
Fossou et al. [42]	Ivory Coast	1251	5–15	8.1% of overweight/obese (10.3% (BMI z-score > + 1SD WHO)) children do not play sport (self-reported)	BMI influenced SBP and DBP in both sexes. Increase in overweight/obesity of children living in higher income municipalities
Nqweniso et al. [43]	South Africa	842	8–13	11.8% did not engage in extracurricular exercise/sport activities (self-reported)	High cardiorespiratory fitness and sport participation negatively associated with overweight/obesity. High sport participation associated with lower HTN risk
Maruf et al. [44]	Nigeria	1517	Mean: Girls 10.2; Boys: 10.5		Obesity indices mediated the association between physical activity (self-reported) and SBP (males: wc, skinfold thickness, and WTHR; females: BMI, wc, and skinfold thickness)
Amponsem-Boateng et al. [38]	Ghana	3165	12–22		Exercise (self-reported) associated with pre-HTN and HTN. Exercising more than 3 times/week reduces pre-HTN and HTN by 20%
**Diet/nutrition**					
Amponsem-Boateng et al. [38]	Ghana	3165	12–22		Homemade foods (self-reported) reduce the odds of pre-HTN or HTN by 21%. Adding extra salt has an increased odds of HTN by 36% and increased the likelihood of pre-HTN by 16%
Skokunbi et al. [46]	Nigeria	488	10–19	Majority consumed far higher (for sodium, 80%) or far below (for potassium, 95%) recommendations	58.1% of participants with HTN consumed eggs 4–6 times/week. Fruits (31.4%), vegetables (36.2%), carbonated drinks (19.0%) and puff-puff (deep fried dough made from refined white flour, sugar, salt, nutmeg and yeast) (48.6%) were less consumed by HTN participants. About 39% of adolescents with HTN add table salt to their already prepared foods
Ayogu and Nwodo [47]	Nigeria	401	10–19	Overweight 3.2% (BMI z-score > + 2SD WHO)	Those who skipped meals had almost twofold higher risk of HTN with impaired fasting capillary glucose
Gewa et al. [39]	Kenya	390	10–12		The proportion of children with HTN was 1.42-fold greater among children with high frequency of consumption of chips/crisps compared with children with lower frequency of consumption
Du Plessis et al. [48]	South Africa	140	13–17	21.5% had a stunted nutrition status	Homocysteine (amino acid found in animal protein) associated with BP. Homocysteine tertiles and BP categories indicates that those in the highest and lowest homocysteine tertiles had a higher risk HTN than those in the middle tertile
**Birth weight / maternal**					
Kaze et al. [49]	Cameroon	80	5–10	26.2% had a low birth weight (< 2500 g) (self-reported)	9.5% of those with low birth weight had HTN
Redjala et al. [50]	Algeria	3562	6–18	22.7% with birthweight < 1500 g had HTN (self-reported)	Pre-HTN and HTN associated with gestational age > 36 weeks, early birth, reduced birth weight, and shorter duration of breastfeeding
Boerstra et al. [51]	South Africa	189	3–6	50% of offspring were born to mothers with HFDP. HFDP-exposed children had a higher rate of preterm birth (< 37 weeks), a higher mean birthweight z-scores and more likely to be born large for gestational age	Maternal hyperglycaemia was not associated with offspring BP (adjusted for offspring age, height and sex)
Syoum et al. [52]	Ethiopia	252	Newborn	Mothers with HTN—20.7% babies had low birth weight, 20.7% were preterm, 10.2% had ICU admissions	Teenage pregnancies were a predictor of maternal complication
De Smidt et al. [53]	South Africa	Controls: 146; cases: 352	5	Children exposed to maternal smoking and alcohol consumption during pregnancy	In utero exposure to alcohol and nicotine was significantly associated with right cIMT measurements. The odds of having a higher than 0.365 mm right cIMT was 1.78 times greater for an exposed child compared to controls
**Interventions**					
Author	**Country**	***n***	**Age range (years)**	**Intervention**	**Effectiveness of intervention**
MOmoniyi et al. [45]	Ghana	78	9–12	Physical activity	Ampe exercise program—body weight (0.31%) and BMI (0.58%) decreased, SBP (3.15%), DBP (1.92%) and heart rate (2.13%) improved after intervention
Elseifi et al. [20]	Egypt	643	12–14	Diet	Skipping breakfast was higher among students with overweight, obesity and increasing BP. Effective in increasing the frequency of healthy breakfast

Abbreviations: *n*, number of participants; BP, blood pressure; HTN, hypertension; EBP, elevated blood pressure; SBP, systolic blood pressure; DBP, diastolic blood pressure; cIMT, carotid intima media thickness; wc, waist circumference; BMI, body mass index; WTHR, waist circumference to height ratio; CVD, cardiovascular disease; DM, diabetes mellitus; BSA, body surface area; ICU, intensive care unit; HDL-c, high density lipoprotein cholesterol; HFDP, hyperglycaemia first detected in pregnancy; WHO, World Health Organisation; CDC, Centre for Disease Control and Prevention; SD, standard deviation

Linking closely with overweight/obesity is the prevalence of *physical inactivity* [38, 41–45], with alarmingly high levels being reported in a South African study by Joubert et al. [41]. In this study, *n* = 109 children aged 8–13 years were included and 39.0% of these children self-reported being physically inactive [41]. Hypertensive children in this study were also more likely to be overweight/obese, but only if they did not meet physical activity recommendations [41]. In a cluster-RCT with *n* = 853 children aged 8–13 years in eight primary schools in Port Elizabeth, South Africa, cardiorespiratory fitness, sport participation, BMI, and BP were assessed at baseline and after a physical activity intervention [43]. The authors showed that high cardiorespiratory fitness and high sport participation were negatively associated with overweight/obesity, while high sport participation was also associated with lower risk for hypertension [43]. The Ampe (a Ghananian house-hold recreational game that includes a combination of physical workout and social bonding) exercise programme implemented in Ghana for instance, proved effective as a paediatric obesity household intervention that provided the impetus for active lifestyles to reduce BP [45]. Longitudinally, those who participated in the physical activity intervention were less likely to become overweight/obese. These findings highlight the relationship between physical activity and body weight.

With regard to *diet* [20, 38, 39, 46–48], data from the studies included in this review suggest that sodium intake is a factor of concern. In the Ghanian national cross-sectional study done by Amponsem-Boateng et al., it was shown that adding extra salt to a meal increases the odds of developing hypertension by 36.0% and by 16.0% for pre-hypertension [38]. In a Nigerian study including *n* = 488 children aged between 10–19 years, the majority consumed far higher levels of sodium and/or far lower levels of potassium than what is recommended with about 39.0% of adolescents with hypertension adding table salt to their already prepared foods [46]. These findings are concerning as the intake of sodium may be set to increase as the African continent undergoes considerable urbanisation.

Early life exposure to unhealthy environmental factors and maternal risk factors [49–53] are linked to an increased risk for elevated BP in children in various African settings. These factors, discussed in the next few paragraphs include among others, hypertension, hypertensive disorders of pregnancy, hyperglycaemia, smoking, alcohol use, overweight/obesity, physical inactivity, and low socioeconomic status [49–53]. Exposure to adverse maternal factors such as smoking, alcohol consumption, and micronutrient deficiency may have a detrimental effect on cardiovascular system development through DNA methylation [54–56].

De Smidt et al. investigated whether there is a link between maternal smoking (nicotine exposure) and alcohol consumption during pregnancy and carotid intima-media thickness (cIMT) in 5-year-old South African children from a low-income environment [53]. The main finding of the study was that exposure to both alcohol and nicotine, maternal adiposity, and male sex of the offspring were associated with an increase in right cIMT at 5 years of age [53]. In contrast, another study in South African children [54], this time from Soweto, a township of Johannesburg, showed that hyperglycaemia first detected in pregnancy was not associated with BP in offspring aged 3–6 years. Regardless of this observation, the prevalence of elevated BP in these children was alarming (49.7%) and warrants further investigations into contributing factors, particularly in low socioeconomic environments to improve detection and develop appropriate interventions.

Recently, a hospital-based cross-sectional study in the Tigray region of Ethiopia assessed foetal and maternal outcomes and associated factors in mothers with hypertensive disorders during pregnancy [52]. A large proportion of the study population (66%) was from rural areas, with rural residence, together with ante- and intrapartum-onset hypertensive disorders of pregnancy later identified as one of the predictors of perinatal complications [52]. More than half of the newborns from hypertensive mothers had adverse outcomes which included among others, low birth weight (20.7%) [52]. This observation highlights the importance of taking into consideration the impact rural versus urban settings have on maternal and subsequently offspring health outcomes, both short and long term, specifically for cardiovascular disease development.

A cross-sectional study in Cameroon explored associations of birth weight with BP and kidney function as assessed by glomerular filtration rate and proteinuria in *n* = 80 children (aged 5–10 years) [49]. The study population was stratified according to low birth weight (< 2500 g), normal birth weight (2500–3999 g), and high birth weight (≥ 4000 g). When focusing on BP, 9.5% of the low birth weight children had hypertension, while 4.4% of the normal weight children had elevated BP [49]. In seven children with proteinuria, 19.0% had low birth weight [49]. Overall, there was a trend towards a negative association between birth weight and BP and kidney function, although not statistically significant.

The shift from rural to urban settings and associated lifestyle changes have a large impact on cardiometabolic risk factors, not only in adults, but in children, although recent data from Africa is scant. A large cross-sectional study focused on school and college-based healthy children (aged 6–18 years) in a suburban area west of Algiers, North Africa [50]. The prevalence of hypertension increased with age, 8.7% for 6–10-year-old children and the highest prevalence (15.6%) was recorded for those older than 15 years of age [50]. In addition, for both prehypertension and hypertension the prevalence increased with higher BMI and was the highest in obese children, 26.8% and 32.3%, respectively [50]. Time spent engaging in sedentary behaviour such as watching television, internet and electronic gaming was associated with prehypertension [50]. Other population characteristics associated with either prehypertension, hypertension or both included parental history of hypertension or diabetes and maternal and postnatal factors such as gestational age, early term delivery, lower birth weight and shorter breastfeeding periods. More comprehensive studies are needed to inform preventative strategies to tackle risk factors associated with the rising burden of childhood hypertension in Africa.

## Current Challenges and Priorities

### Methodological Issues

The detection of hypertension in children and adolescents is highly dependent on the proper measurement of BP in research and clinical practice settings and is affected when methodological aspects are not optimal. While BP is a vital sign and a standard diagnostic tool in clinical practice [57], measurement of BP in children and follow-up is often neglected in clinical practice [57]. When measured, several errors have been reported which seem to have universal characteristics for both children and adults. Some of these errors are related to observer bias or mistakes due to a lack of proper training, the patient’s behaviour or experience during measurements, while some errors are inherent to the device algorithms due to proprietary rights from manufacturers who are unwilling to disclose algorithm coefficients and factors used in their algorithm or transfer function development. While the effects of risk factor exposure in children are less extensively known, other factors such as temperature [58], noise exposure [59], pollutants [60] and/or discomfort related to a lack of privacy during the measurement can contribute to errors in BP measurements [61, 62]. In Africa, the methodological issues related to BP measurement, diagnosis and treatment are undeniably more challenging. These challenges include (among others) the limited access to and affordability of paediatric validated BP devices, accredited calibration centres, the lack of African specific nomograms for childhood and adolescent BP, and the non-existence of proper treatment algorithms for the massive population diversity on the African continent. In this section, the methodological issues will be briefly discussed under four main categories namely the patient (“The Patient”), the observer (“The Observer”), BP devices (“The Device”), as well as the protocol and guidelines (“The Protocol and Guidelines”).

#### The Patient

Measuring BP accurately largely depends on the cooperation of the patient and their understanding of the conditions under which the measurements should be taken. Barriers specific to the patient can include language, age, body size, mood, culture, and illness or circumstances in which BP is measured. In Africa, over 2000 languages are spoken. Furthermore, the rural population comprise more than half of Africa [63], which poses several barriers in terms of education and communication. Basic BP measurement instructions (i.e. back supported and feet flat on the ground without talking) provided by health care professionals are difficult to understand by a large proportion of individuals and may be even more challenging in the childhood population, when local or indigenous languages are mostly learned. The language barrier is accompanied by age, since younger children (e.g. under 5 years) find it more challenging to follow such instructions. These younger age groups are also less likely to keep still for prolonged periods while BP is measured and are often restless with their feet, especially when the feet are not properly supported due to the use of an adult sized chair for a child. Larger children or adolescents on the other hand, provide a challenge in terms of BP cuff size. Some manufacturers do not offer a large range in cuff sizes specific to the paediatric population and observers then need to improvise by using adult-sized cuffs, which may contribute to under- or over- estimatation of BP when the cuff is either too big or too small [64]. Children can be unpredictable in how they tolerate BP measurement and their temperament can influence the BP readings when a child is moody and does not enjoy the experience of the measurements, even more so for ambulatory BP monitoring (ABPM) [65]. Due to several geopolitical issues in many African countries, there also exist cultural conflict when the observer and patient do not share compatible cultural beliefs or tolerability for certain ethnic groups [66]. This is a reality on the African continent and ethnic discord has contributed to many challenges in the clinical spheres of treatment and healing due to the spillover of political favouritism in African countries [67]. Therefore, in some cultures in Africa it works best to measure BP in a group of children and not in isolation, to reduce the risk of fear and stigma when participating in research studies, although this is not what clinical guidelines from developed countries propose. Lastly, in certain disease conditions when it is not possible to measure a child’s BP in the optimal sitting position, there are several challenges due to divergent criteria available in the literature and the lack of clinical practice guidelines in Africa for detecting primary hypertension in children and adolescents.

#### The Observer

The nurse, medical specialist, clinician, or researcher is referred to as the observer taking the BP measurement. Similar barriers, as for patients, are evident in the clinical setting whereby nurses or clinicians have to measure BP. However, apart from language differences, managing a very young child (position, cuff size, cuff position and arm selection, removing thick or tight fitting clothing, etc.), and device preference (manual sphygmomanometer, auscultatory or automated (oscillometry) may lead to several observer specific barriers that lead to errors in BP measurements. While proper training for BP measurement can not be more emphasised, deviations from protocols are a given in most busy hospitals or clinical settings whereby nurses or doctors do not have enough time to perform proper BP measurements, or in some cases not at all [68]. The real-life environment for medical staff is a challenge by itself in terms of multitasking, time management, the availability of equipment, and the location of practice (e.g. crowded and noisy clinics with limited space but large patient influx in the public sector compared to more controlled environments in the private sector) [69, 70]. An international survey indicated that Africa has the greatest workforce burden for the number of paediatricians per 100,000 children and adolescents under 18 years with a reported median ratio of 0.8 (IQR: 0.4–2.6) [71]. As a result of such rushed environments and circumstances, the standard protocol for accurate BP measurement is overlooked and often no rest period is given to patients before the first BP measurement is taken, while in most cases no further measurements are performed and clinical decisions are based on one non-standardised BP value. In addition, talking remains one of the greatest human errors in BP measurement. When a patient talks while the cuff inflates and deflates, BP is directly affected. Similarly, when nurses, doctors or other medical specialists have discussions during the BP measurement, BP errors are unavoidable.

#### The Device

The validation of BP devices is one of the persistent global methodological challenges in the hypertension sphere [60]. Several BP devices have been validated for clinical use, while no BP device has been validated in Africa or specifically in any African population group. Validating BP devices in Africa is a priority area since there is a lack of data available that illustrates whether BPs are under- or overestimated by BP devices validated elsewhere. The propriatry nature of automated BP device algorithms, limits the ability to distinctively identify the factors that influence BP readings for a specific manufacturer’s device model(s). It is therefore unknown whether such algorithms account for biological and ethnic differentiation between population groups from various parts of the world [72]. With the majority of validation studies being outdated due to the recent updates of the international validation protocols, such studies were also in violation of the validation protocols as a result of incomplete reporting of essential information [73]. In addition to devices being validated, the regular calibration of BP devices seems neglected. Calibration of aneroid devices is essential for accurate BP measurement, while electronic devices need to be serviced annually by accredited centres to assure BP accuracy over time [70]. In addition to aneroid, automated or semi-automatic oscillerometric devices for clinic use, there are also device related concerns in Africa specific to 24 h ABPM, home BP monitoring and cuffless or wearable technology. While use of ABPM is optimal for clinical practice and in special conditions in paediatrics due to its benefits (i.e. good reproductibility, detection of white-coat and masked hypertension [74], demonstration of BP variability and dipping patterns [75]), access and affordibility are two of the main limitations to use in African and other developing countries. Ambulatory BP monitoring is used in Africa especially in private hospitals and clinical research settings, however, there remains a major risk of device loss and damages of such devices. Home BP monitoring is slightly more challenging due to limited time for proper patient training on the use of the device, the availability of home BP monitors in clincal practice or cost to patients to purchase a validated BP device for home use. Connectivity is another challenge when BP readings need to be sent to a central patient registry for BP telemonitoring, especially in deep-rural areas with limited mobile network infrastructure or high cost for mobile data or wireless connectivity. Although wearable technology is expanding globally, and access is available in African countries, such devices are not currently of much clinical use due to poor reliability and/or accuracy, and lack of validated wearable or cuffless devices [76]. Another critical device-related criteria for accurate BP measurement is the cuff size and placement [77]. In paediatrics, upper arm circumferences are ranging vastly from extremely thin to adult size. Unfortunately, not all BP manufacturers provide a sufficient variety of cuff sizes. Using the wrong cuff size can provide massive errors in BP measurements. Independent of the type of device, the cuff size and placement remain one of the biggest pitfalls in BP measurement accuracy.

#### The Protocol and Guidelines

Even among the internationally recognised hypertension guidelines there are various discrepencies contributing to confusion and non-standardised methodology when applied in Africa. Especially in low resource settings where time is limited and the clinician to patient ratio is dramatically skewed, studies reported high numbers of paediatric patients not screened for hypertension in primary care. The main reason is the absence of risk factors for hypertension (especially secondary hypertension), increasing the potential to under-diagnose primary hypertension [68]. A study in South Africa has shown that using the mean of the lowest three of five BP measurements in young (5–9 years old) children [27••], almost 40.0% of the children had abnormal BP based on the 2017 American Academy of Pediatric Clinical Practice Guideline (2017, AAP CPG) [77], albeit at a single office visit. The number of BP measurements that should be performed at a single visit in the clinic differs in the currently available guidelines from other countries. Some guidelines advise on discarding the first measurement and use the mean of the second and third [78], while others support using the mean of two measurements or one measurement and only repeated twice if BP was elevated [77]. As expected, BP will drop with consecutive measurements until tolerance is saturated, however, no consensus exist currently on the number of BP measurements required for clinical use. For hypertension status confirmation, a second and third visit is required. But in reality, due to constraints in the primary health care environment, BP is often measured once to determine BP status or not at all in the case of otherwise healthy children or adolescents. Guidelines recommend to allow a 3–5-min rest before and a 1–2-min interval when performing repeated BP measurements with automated BP devices. A rest period prior to BP measurement is necessary to calm the patient and to ensure that all conditions are met to acquire an accurate BP reading. However, a study reported that negligible differences were observed between 30-s and 60-s intervals and indicated that shorter time intervals are more feasible in clinical practice [79], while guidelines still recommend 1-min intervals. Since the recommendation of automated BP devices [80], due to its limited margin of human error, some task groups and authors have indicated that observer training and re-training is non-essential when making use of automated or semi-automated devices. While human errors in terms of auscultation is absent for oscillometric devices, human error is still involved in the selection of cuff sizes, environmental conditions in which BP is measured, proper patient counselling and positioning, adherence to recommended guidelines in terms of resting periods and time intervals in-between measurements and finally recording BP values correctly to a patient record.

The variety of hypertension guidelines in paediatrics is also a potential pitfall for African countries (and others) that do not have their own nomograms for BP in children and adolescents [81••]. Several childhood BP guidelines are in use to allow for the detection of high-risk children and adolescents. These include, among others, the Report on the Diagnosis, Evaluation, and Treatment of High Blood Pressure in Children and Adolescents (NHBPEP) (2004 Fourth Report) [82], 2017 AAP CPG [77] and the European Society of Hypertension (2016 ESH) [78]. These age, sex and body height specific BP guidelines originate from high-income countries (HICs). Several population-based studies in several parts of Asia, Europe, Northern Africa and the United States of America (USA), have prospectively examined the usefulness of these HICs childhood clinical practice guidelines in identifying high-risk children and adolescents [83, 84]. Certainly, adult hypertension guidelines are frequently applied in LMIC settings illustrating that a HIC BP guidelines can perform in a LMIC adult population. A recent mixed cross-section longitudinal study conducted in South Africa highlighted that when the three most applied international childhood clinical practice guidelines (2004 Fourth report; 2016 ESH and 2017 AAP) were used to detect hypertension in an African paediatric cohort, a varied hypertension prevalence was reported [81••]. Consistent with the findings from numerous recent cross-sectional studies conducted worldwide [84–86], the 2017 AAP CPG identified a greater hypertension prevalence and a decrease in the number of children and adolescents with pre-hypertension showing a concomitant upward trend in the prevalence of hypertension [81••, 83, 87]. The most prominent reason for the observed disparities is the notable differences seen in the BP charts or nomograms. The 2017 AAP CPG guideline excludes obese or overweight children and adolescents. In an era where childhood obesity is a growing concern, in conjunction with the fact that body composition (i.e. obesity) is a significant cause for the development of primary hypertension, a population that includes obese or overweight youth will render an heightened overall hypertension prevalence. Another prominent reason for the varied prevalence may be due to the linear growth of the population as nomograms are body height specific. When comparing the various paediatric BP guidelines, the 2017 AAP CPG for example shows a much broader BP range for shorter individuals, while the gaps tends to decrease for taller individuals [77]. Results from the study by Craig et al. (2022), showed that the African paediatric cohort was on average shorter than that expected according to age- and sex-matched growth charts listed by the WHO [81••, 88]. Another recent study carried out on *n* = 32 248 Zimbabwean children also reported that the African paediatric cohort were shorter and weighed less in comparison to WHO growth charts [89]. Therefore, African youth of a shorter stature may have a lower threshold for elevated BP due to being shifted into a low height percentile. As a result, applying the simple adoption of childhood BP nomograms from a HIC may not be best suited for LMICs with diverse population characteristics. These guidelines may therefore introduce unpredicted bias in evaluating childhood BP resulting in a significant over or under-estimation of the BP status. The need to explore country or region-specific BP guidelines has therefore gained momentum considering the increasing childhood hypertension burden in Africa alone.

## Clinical Perspectives—Current Challenges and Priorities

Despite the availability of international BP nomograms [77, 78, 90–92] and proposed paediatric hypertension guidelines [77, 78, 93–95], the applicability thereof in Africa remain uncertain. Available nomograms for childhood BP are from countries with the lowest to no number of citizens from African ancestry compared to Africa. These countries include the USA [77], China [93], Japan [95] and Western European countries [78], while other countries such as Canada [94] and India [96] have their own proposed criteria for detecting hypertension in children and adolescents. While these are all essential documents and useful to paediatricians, paediatric specialists and epidemiologists, there remains much work to be done to define BP nomograms in Africa and to determine the usefulness of international paediatric hypertension guidelines in the African region. Notwithstanding the support for efforts to sensitise the need for region-specific normative data, expert groups from all parts of the world should aim to forge globalised guidelines for the detection and management of primary hypertension in paediatrics.

Another concern is that there are no recent data informing the use of antihypertensive agents in children and adolesents in Africa. Additionally, there are no appropriate data to assess the long-term effectiveness of treatment of high BP in children or adolescents with pharmacological interventions that may have the ability to reduced BP and adverse health outcomes in later life. An Africa-centered collaborative effort to intensify peadiatric hypertensive studies is urgently needed to address this knowledge gap on the African continent.

## Conclusion

Despite several efforts to understand childhood onset of hypertension in Africa, remaining challenges include limited access to healthcare, inadequate resources for screening and diagnosis, and poor awareness among caregivers and healthcare professionals regarding the importance of early detection and management. Overall, the studies addressing childhood hypertension have emphasised that research, resources, and policies need to be upscaled to address this burgeoning public health concern.

## References

[1] Global Action Plan for the prevention and control of noncommunicable diseases 2013–2020. https://apps.who.int/iris/bitstream/handle/10665/94384/9789241506236_eng.pdf;jsessionid=993FD743F06FE0D93E3183921550AD73?sequence=1.

[2] Kagura J, Adair LS, Musa MG, Pettifor JM, Norris SA (2015). Blood pressure tracking in urban black South African children: birth to twenty cohort. BMC Pediatr.

[3] Juhola J, Magnussen CG, Viikari JS, Kahonen M, Hutri-Kahonen N, Jula A (2011). Tracking of serum lipid levels, blood pressure, and body mass index from childhood to adulthood: the Cardiovascular Risk in Young Finns Study. J Pediatr.

[4] Webber LS, Cresanta JL, Voors AW, Berenson GS (1983). Tracking of cardiovascular disease risk factor variables in school-age children. J Chronic Dis.

[5] Nelson MJ, Ragland DR, Syme SL (1992). Longitudinal prediction of adult blood pressure from juvenile blood pressure levels. Am J Epidemiol.

[6] Pieters M, Vorster HH (2008). Nutrition and hemostasis: a focus on urbanization in South Africa. Mol Nutr Food Res.

[7] Chen X, Wang Y (2008). Tracking of blood pressure from childhood to adulthood: a systematic review and meta-regression analysis. Circulation.

[8] Litwin M, Feber J (2020). Origins of primary hypertension in children: early vascular or biological aging?. Hypertension.

[9] Ehret GB, Bakris B, Forman J. Genetic factors in the pathogenesis of hypertension. Access mode: https://www.Uptodate.com/contents/genetic-factors-in-the-pathogenesis-of-hypertension. Last time updated: Dec, 2017. 13.

[10] Oikonen M, Tikkanen E, Juhola J, Tuovinen T, Seppälä I, Juonala M (2011). Genetic variants and blood pressure in a population-based cohort: the Cardiovascular Risk in Young Finns study. Hypertension.

[11] Howe LD, Parmar PG, Paternoster L, Warrington NM, Kemp JP, Briollais L (2013). Genetic influences on trajectories of systolic blood pressure across childhood and adolescence. Circ Cardiovasc Genet.

[12] Raitakari OT, Juonala M, Rönnemaa T, Keltikangas-Järvinen L, Räsänen L, Pietikäinen M (2008). Cohort profile: the cardiovascular risk in Young Finns Study. Int J Epidemiol.

[13] Fraser A, Macdonald-Wallis C, Tilling K, Boyd A, Golding J, Davey Smith G (2013). Cohort Profile: the Avon Longitudinal Study of Parents and Children: ALSPAC mothers cohort. Int J Epidemiol.

[14] Williams LA, Evans SF, Newnham JP (1997). Prospective cohort study of factors influencing the relative weights of the placenta and the newborn infant. BMJ.

[15] International Monetary Fund: GDP per capita, current prices. https://www.imf.org/external/datamapper/NGDPDPC@WEO/OEMDC/ADVEC/WEOWORLD (2021).

[16] Hassan NE, El Shebini SM, El-Masry SA, Ahmed NH, Alia MM, El-Saeed GSM (2019). Association between dietary sodium, calcium, saturated fat and blood pressure in obese Egyptian adolescents. Gaz Egypt Paediatr Assoc.

[17] El-Koofy N, Soliman H, Elbarbary MA, Garhy ASE, Sheba M, Fouad H (2020). Use of anthropometry versus ultrasound for the assessment of body fat and comorbidities in children with obesity. J Pediatr Gastroenterol Nutr.

[18] Lule SA, Namara B, Akurut H, Muhangi L, Lubyayi L, Nampijja M (2019). Are birthweight and postnatal weight gain in childhood associated with blood pressure in early adolescence? Results from a Ugandan birth cohort. Int J Epidemiol.

[19] Munter P, He J, Cutler JA, Wildman RP, Whelton PK (2004). Trends in blood pressure among children and adolescents. JAMA.

[20] Elseifi OS, Abdelrahman DM, Mortada EM (2020). Effect of a nutritional education intervention on breakfast consumption among preparatory school students in Egypt. Int J Public Health.

[21] Hassan NE, El Ashmawi AA, El-Masry SA, Zarouk WA, Mira MF, El-Saeed GSM (2019). Metabolic syndrome in a sample of Egyptian adolescent girls and its association with apolipoprotein E. J Paediatr Child Health.

[22] •• Muyumba EK, Nkulu DN, Mukeng CK, Musung JM, Kakoma PK, Kakisingi CN, et al. Oscillometric blood pressure by age and height for non overweight children and adolescents in Lubumbashi, Democratic Republic of Congo. BMC Cardiovasc Disord. 2018;18(1):9. 10.1186/s12872-018-0741-4. **This paper is of significance since it is currently the only study from Africa that determined BP percentiles (50, 90 and 95) for age and height in more than 7,000 children and adolescents aged 3 to 17 years in the DRC. For the most part, these thresholds were higher in comparison to BP percentiles from other countries, although comparisons with European and American percentiles were absent.** 10.1186/s12872-018-0741-4 PMC5775618 29351738

[23] •• Crouch SH, Soepnel LM, Kolkenbeck-Ruh A, Maposa I, Naidoo S, Davies J, et al. Paediatric hypertension in Africa: a systematic review and meta-analysis. EClinicalMedicine. 2021;43:101229. 10.1016/j.eclinm.2021.101229. **The most recent summary of hypertension prevelance was captured by this systematic review and meta-analysis paper, showing the vast differences in childhood hypertension rates across the African regions and the role of obesity as a key factor contributing to hypertension in children and adolescents.** 10.1016/j.eclinm.2021.101229 PMC8665406 34917909

[24] Noubiap JJ, Essouma M, Bigna JJ, Jingi AM, Aminde LN, Nansseu JR (2017). Prevalence of elevated blood pressure in children and adolescents in Africa: a systematic review and meta-analysis. Lancet Public health.

[25] •• Sani RN, Connelly PJ, Toft M, Rowa-Dewar N, Delles C, Gasevic D, et al. Rural-urban difference in the prevalence of hypertension in West Africa: a systematic review and meta-analysis. J Hum Hypertens. 2022. 10.1038/s41371-022-00688-8. **This paper from West Africa highlight important differences in rural versus urban trends in hypertension prevalence with no specific differences observed between sexes. This analysis was limited to ages ranging from 15 years to above 70 years, and no data available in children.** 10.1038/s41371-022-00688-8 PMC11001577 35430612

[26] Aounallah-Skhiri H, Romdhane HB, Traissac P, Eymard-Duvernay S, Delpeuch F, Achour N (2008). Nutritional status of Tunisian adolescents: associated gender, environmental and socio-economic factors. Public Health Nutr.

[27] •• Kruger R, Kruger HS, Monyeki MA, Pienaar AE, Roux SB, Gafane-Matemane LF, et al. A demographic approach to assess elevated blood pressure and obesity in prepubescent children: the ExAMIN Youth South Africa study. J Hypertens. 2021;39(11):2190–9. 10.1097/HJH.0000000000002917. **This paper in prepubescent children showed important ethnic and sex differences in blood pressure and the high rate (37%) of children with abnormal blood pressure based on the 2017 American Academy of Pediatrics Clincal Practice Guidelines, highlighting the concern for using thresholds from other countries.** 10.1097/HJH.0000000000002917 34620809

[28] •• Afaa TJ, H Seneadza NA, Ameyaw E, Rodrigues OP. Blood pressure profile, prevalence of hypertension and associated familial factors in school children in Accra, Ghana. Niger J Clin Pract. 2022;25(4):386–90. 10.4103/njcp.njcp_1832_21. **This paper illustrated the importance of family history of hypertension in lifestyle interventions to address risk factors for hypertension development from young ages. The potential role of genetic factors in the development of primary hypertension was highlighted.** 10.4103/njcp.njcp_1832_21 35439894

[29] Sungwa EE, Kibona SE, Dika HI, Laisser RM, Gemuhay HM, Kabalimu TK (2020). Prevalence and factors that are associated with elevated blood pressure among primary school children in Mwanza Region. Tanzania Pan Afr Med J.

[30] Oluwayemi IO, Oyedeji OA (2021). A ten-year review of childhood obesity in a teaching hospital, South West Nigeria. Niger J Clin Pract.

[31] Akinbodewa AA, Adejumo AO, Lamidi OA, Adeyemi O (2020). Clustering of Cardiometabolic Risk Factors among Children and Adolescents in a Rural Community in Ondo. Southwest Nigeria J Trop Pediatr.

[32] Letswalo BP, Schmid-Zalaudek K, Brix B, Matjuda EN, Klosz F, Obernhumer N, et al. Cardiometabolic risk factors and early indicators of vascular dysfunction: a cross-sectional cohort study in South African adolescents. BMJ Open. 2021;11(3):e042955. 10.1136/bmjopen-2020-042955. 10.1136/bmjopen-2020-042955 PMC7978086 33737426

[33] Ukoh UC, Ujunwa FA, Muoneke UV, Manyike PC, Okike CO, Ibe BC (2020). Oscillometric blood pressure profile of adolescent secondary school students in Abakaliki metropolis. Ann Afr Med.

[34] Gomwe H, Seekoe E, Lyoka P, Marange CS (2022). Blood pressure profile of primary school children in Eastern Cape province, South Africa: prevalence and risk factors. BMC Pediatr.

[35] Raphadu TT, Staden MV, Dibakwane WM, Monyeki KD (2020). A non-invasive investigation into the prevalence of higher than normal blood pressure, hypertension and the association between blood pressure and body weight in male and female adolescents in the Polokwane local municipality, Limpopo-South Africa: a cross-sectional study. Children (Basel).

[36] Matjuda EN, Engwa GA, Letswalo PB, Mungamba MM, Sewani-Rusike CR, Nkeh-Chungag BN (2020). Association of hypertension and obesity with risk factors of cardiovascular diseases in children aged 6–9 years old in the Eastern Cape Province of South Africa. Children (Basel).

[37] Mokgwathi M, Mwita JC (2020). Prevalence of hypertension and selected cardiovascular risk factors among adolescents in selected rural and urban secondary schools in Botswana. Cardiovasc J Afr.

[38] Amponsem-Boateng C, Oppong TB, Zhang W, Boakye-Yiadom J, Wang L, Acheampong K, et al. Screening of hypertension, risks, knowledge/awareness in second-cycle schools in Ghana. A national cross-sectional study among students aged 12–22. J Hum Hypertens. 2022;36(4):405–15. 10.1038/s41371-021-00502-x. 10.1038/s41371-021-00502-x 33790406

[39] Gewa CA, Onyango AC, Opiyo RO, Gittelsohn J, Cheskin LJ (2022). Patterns and predictors of elevated blood pressure and hypertension among primary school children in urban Kenya. J Hypertens.

[40] Kansiime S, Webb EL, Kusemererwa S, Lule SA, Niwaha AJ, Seeley J (2022). Blood pressure levels among children in rural Uganda: results from 1913 children in a general population survey. J Hum Hypertens.

[41] Joubert N, Walter C, du Randt R, Aerts A, Adams L, Degen J (2021). Hypertension among South African children in disadvantaged areas and associations with physical activity, fitness, and cardiovascular risk markers: A cross-sectional study. J Sports Sci.

[42] Fossou AF, Ahui Bitty ML, Coulibaly TJ, Bataï NF, Touré MF, Zahé KYAS (2020). Prevalence of obesity in children enrolled in private and public primary schools. Clin Nutr ESPEN.

[43] Nqweniso S, Walter C, du Randt R, Aerts A, Adams L, Degen J (2020). Prevention of overweight and hypertension through cardiorespiratory fitness and extracurricular sport participation among South African schoolchildren. Sustainability.

[44] Maruf FA, Odetunde MO, Okonkwo PU (2020). Association between physical activity level and blood pressure: varied and graded mediating effects of obesity indices in schoolchildren. Cardiol Young.

[45] MOmoniyi MM, Afrifa D, Asamoah MA, Sarpong P, Sarpong E, Appiah PO, Akoto F. "AMPE" exercise programme has positive effects on anthropometric and physiological parameters of school children: a pilot study. Ethiop J Health Sci. 2020;30(1):143–6. 10.4314/ejhs.v30i1. 10.4314/ejhs.v30i1.18 PMC7036453 32116443

[46] Shokunbi OS, Ukangwa NA (2021). Relationship of blood pressure status, dietary factors and serum electrolytes of in-school adolescents in Ilishan-Remo, Ogun State. Nigeria Afr Health Sci.

[47] Ayogu RNB, Nwodo CJ. Epidemiological characteristics of hypertension, impaired fasting capillary glucose and their comorbidity: a retrospective cross-sectional population-based study of rural adolescents in Southeast Nigeria. BMJ Open. 2021;11(5):e041481. 10.1136/bmjopen-2020-041481. 10.1136/bmjopen-2020-041481 PMC8103371 33952534

[48] du Plessis JP, Nienaber-Rousseau C, Lammertyn L, Schutte AE, Pieters M, Kruger HS (2021). The relationship of circulating homocysteine with fibrinogen, blood pressure, and other cardiovascular measures in African adolescents. J Pediatr.

[49] Kaze FF, Nguefack S, Asong CM, Assob JCN, Nansseu JR, Kowo MP (2020). Birth weight and renal markers in children aged 5–10 years in Cameroon: a cross-sectional study. BMC Nephrol.

[50] Redjala O, Sari-Ahmed M, Cherifi M, Smati L, Benhassine F, Baghriche M (2021). Children hypertension in Northern Africa. Am J Cardiovasc Dis.

[51] Boerstra BA, Soepnel LM, Nicolaou V, Kolkenbeck-Ruh A, Kagura J, Ware LJ (2022). The impact of maternal hyperglycaemia first detected in pregnancy on offspring blood pressure in Soweto. South Africa J Hypertens.

[52] Syoum FH, Abreha GF, Teklemichael DM, Chekole MK (2022). Fetomaternal outcomes and associated factors among mothers with hypertensive disorders of pregnancy in Suhul Hospital, Northwest Tigray. Ethiopia J Pregnancy.

[53] De Smidt JJA, Odendaal HJ, Nel DG, Nolan H, Du Plessis C, Brink LT (2021). In utero teratogen exposure and cardiometabolic risk in 5-year-old children: a prospective pediatric study. J Matern Fetal Neonatal Med.

[54] Rauschert S, Melton PE, Burdge G, Craig JM, Godfrey KM, Holbrook JD (2019). Maternal smoking during pregnancy induces persistent epigenetic changes into adolescence, independent of postnatal smoke exposure and is associated with cardiometabolic risk. Front Genet.

[55] Naik VD, Lee J, Wu G, Washburn S, Ramadoss J (2022). Effects of nutrition and gestational alcohol consumption on fetal growth and development. Nutr Rev.

[56] Mandal C, Halder D, Jung KH, Chai YG (2017). Gestational alcohol exposure altered DNA methylation status in the developing fetus. Int J Mol Sci.

[57] O'Brien E, Stergiou GS, Turner MJ (2018). The quest for accuracy of blood pressure measuring devices. J Clin Hypertens (Greenwich).

[58] Zhao H, Jivraj S, Moody A (2019). 'My blood pressure is low today, do you have the heating on?' The association between indoor temperature and blood pressure. J Hypertens.

[59] D'Souza J, Weuve J, Brook RD, Evans DA, Kaufman JD, Adar SD (2021). Long-term exposures to urban noise and blood pressure levels and control among older adults. Hypertension.

[60] Huang M, Chen J, Yang Y, Yuan H, Huang Z, Lu Y. Effects of ambient air pollution on blood pressure among children and adolescents: a systematic review and meta-analysis. J Am Heart Assoc. 2021;10(10):e017734. 10.1161/JAHA.120.017734. 10.1161/JAHA.120.017734 PMC8200690 33942625

[61] Hardy ST, Urbina EM (2021). Blood pressure in childhood and adolescence. Am J Hypertens.

[62] Badihian N, Riahi R, Qorbani M, Motlagh ME, Heshmat R, Kelishadi R (2020). The associations between noise annoyance and psychological distress with blood pressure in children and adolescents: The CASPIAN-V Study. J Clin Hypertens (Greenwich).

[63] Bank TW. Rural population in Africa, ID: SP.RUR.TOTL. 2021: Washington, D.C., United States.

[64] Varney EJ, Van Drunen AM, Moore EF, Carlin K, Thomas K (2019). Blood pressure measurement error in children: lessons in measurement reliability. J Nurs Meas.

[65] Hamdani G, Flynn JT, Daniels S, Falkner B, Hanevold C, Ingelfinger J (2019). Ambulatory blood pressure monitoring tolerability and blood pressure status in adolescents: the SHIP AHOY study. Blood Press Monit.

[66] Marshall MG, Elzinga-Marshall G, Global report 2017: Conflict, governance and state fragility. 2017. https://www.systemicpeace.org/globalreport.html.

[67] Beiser-McGrath J, Müller-Crepon C, Pengl Y (2021). Who Benefits? How local ethnic demography shapes political favoritism in Africa. British Journal of Political Science.

[68] Bijlsma MW, Blufpand HN, Kaspers GJ, Bökenkamp A (2014). Why pediatricians fail to diagnose hypertension: a multicenter survey. J Pediatr.

[69] Elias MF, Goodell AL (2021). Human errors in automated office blood pressure measurement: still room for improvement. Hypertension.

[70] Padwal R, Campbell NRC, Schutte AE, Olsen MH, Delles C, Etyang A (2019). Optimizing observer performance of clinic blood pressure measurement: a position statement from the Lancet Commission on Hypertension Group. J Hypertens.

[71] Harper BD, Nganga W, Armstrong R, Forsyth KD, Ham HP, Keenan WJ, Russ CM. Where are the paediatricians? An international survey to understand the global paediatric workforce. BMJ paediatrics open. 2019;3(1): e000397. 10.1136/bmjpo-2018-000397. 10.1136/bmjpo-2018-000397 PMC6361365 30815583

[72] Sharman JE, O'Brien E, Alpert B, Schutte AE, Delles C, Hecht Olsen M (2020). Lancet Commission on Hypertension group position statement on the global improvement of accuracy standards for devices that measure blood pressure. J Hypertens.

[73] Stergiou GS, O'Brien E, Myers M, Palatini P, Parati G, Kollias A, et al. STRIDE BP Scientific Advisory Board. STRIDE BP international initiative for accurate blood pressure measurement: Systematic review of published validation studies of blood pressure measuring devices. J Clin Hypertens (Greenwich). 2019;21(11):1616–22. 10.1111/jch.13710. 10.1111/jch.13710 PMC8030378 31654494

[74] Bo Y, Kwok KO, Chung VC, Yu CP, Tsoi KK, Wong SY (2020). Short-term reproducibility of ambulatory blood pressure measurements: a systematic review and meta-analysis of 35 observational studies. J Hypertens.

[75] Flynn JT, Daniels SR, Hayman LL, Maahs DM, McCrindle BW, Mitsnefes M (2014). Update: ambulatory blood pressure monitoring in children and adolescents: a scientific statement from the American Heart Association. Hypertension.

[76] Stergiou GS, Mukkamala R, Avolio A, Kyriakoulis KG, Mieke S, Murray A (2022). Cuffless blood pressure measuring devices: review and statement by the European Society of Hypertension Working Group on Blood Pressure Monitoring and Cardiovascular Variability. J Hypertens.

[77] Flynn JT, Kaelber DC, Baker-Smith CM, et al. Subcommittee on Screening and Management of High Blood Pressure in Children. Clinical practice guideline for screening and management of high blood pressure in children and adolescents. Pediatrics. 2017;140(3):e20171904. Pediatrics. 2018;142(3):e20181739. 10.1542/peds.2018-1739. 10.1542/peds.2018-1739 30177515

[78] Lurbe E, Agabiti-Rosei E, Cruickshank JK, Dominiczak A, Erdine S, Hirth A (2016). European Society of Hypertension guidelines for the management of high blood pressure in children and adolescents. J Hypertens.

[79] Juraschek SP, Ishak AM, Mukamal KJ, Wood JM, Anderson TS, Cohen ML (2021). Impact of 30- versus 60-second time intervals between automated office blood pressure measurements on measured blood pressure. Hypertension.

[80] Sharman JE, Tan I, Stergiou GS, Lombardi C, Saladini F, Butlin M (2023). Automated ‘oscillometric’ blood pressure measuring devices: how they work and what they measure. J Hum Hypertens.

[81] •• Craig A, Ware LJ, Mapanga W, Norris SA. A comparison of paediatric hypertension clinical practice guidelines and their ability to predict adult hypertension in an African birth cohort. J Hum Hypertens. 2022. 10.1038/s41371-022-00709-6. **This was the first study in Africa to determine the predictive value of childhood blood pressure by using commonly used paediatric clinical practice guidelines in the African context. This paper showed that the performance of guidelines developed elsewhere were not optimal in predicting adult hypertension, with the 2017 AAP CPG showing the most acceptable level of performance.**

[82] National High Blood Pressure Education Program Working Group on High Blood Pressure in Children and Adolescents The fourth report on the diagnosis, evaluation, and treatment of high blood pressure in children and adolescents. Pediatrics. 2004;114:555–576. 15286277

[83] Du T, Fernandez C, Barshop R, Chen W, Urbina EM, Bazzano LA (2019). 2017 Pediatric hypertension guidelines improve prediction of adult cardiovascular outcomes. Hypertension.

[84] Yang L, Kelishadi R, Hong YM, Khadilkar A, Nawarycz T, Krzywińska-Wiewiorowska M (2019). Impact of the 2017 American Academy of Pediatrics Guideline on Hypertension Prevalence Compared With the Fourth Report in an International Cohort. Hypertension.

[85] Condren M, Carter J, Mushtaq N, Puckett S, Kezbers K, Sabet S (2019). The Impact of new guidelines on the prevalence of hypertension in children: a cross-sectional evaluation. J Clin Hypertens.

[86] Dong Y, Song Y, Zou Z, Ma J, Dong B, Prochaska JJ (2019). Updates to pediatric hypertension guidelines: influence on classification of high blood pressure in children and adolescents. J Hypertens.

[87] Al Kibria GM, Swasey K, Sharmeen, Day B. Estimated changes in prevalence and trends of childhood blood pressure levels in the United States after application of the 2017 AAP guideline. Prev Chronic Dis. 2019;16:e12. 10.5888/pcd16.180528. 10.5888/pcd16.180528 PMC6362707 30702999

[88] de Onis M, Onyango AW, Borghi E, Siyam A, Nishida C, Siekmann J (2007). Development of a WHO growth reference for school-age children and adolescents. Bull World Health Organ.

[89] Marume A, Moherndran A, Tinarwo P, Mahomed S (2022). Development of a Zimbabwean child growth curve and its comparison with the World Health Organization child growth standards. Afr J Prim Health Care Fam Med.

[90] Neuhauser HK, Thamm M, Ellert U, Hense HW, Rosario AS. Blood pressure percentiles by age and height from nonoverweight children and adolescents in Germany. Pediatrics. 2011;127(4):e978–88. 10.1542/peds.2010-1290. Epub 2011 Mar 7 PMID: 21382947. 10.1542/peds.2010-1290 21382947

[91] Xi B, Zong X, Kelishadi R, Hong YM, Khadilkar A, Steffen LM (2016). Establishing international blood pressure references among nonoverweight children and adolescents aged 6 to 17 years. Circulation.

[92] Müller I, Smith D, Adams L, Aerts A, Damons BP, Degen J, et al.. Effects of a school-based health intervention program in marginalized communities of port Elizabeth, South Africa (the Kazibantu Study): protocol for a randomized controlled trial. JMIR Res Protoc. 2019;8:e14097. 10.2196/14097. 10.2196/14097 PMC6657454 31298224

[93] Joint Committee for Guideline Revision. 2018 Chinese guidelines for prevention and treatment of hypertension-a report of the revision committee of Chinese guidelines for prevention and treatment of hypertension.J Geriatr Cardiol. 2019;16:182–241. 10.11909/j.issn.1671-5411.2019.03.014. 10.11909/j.issn.1671-5411.2019.03.014 PMC6500570 31080465

[94] Rabi DM, McBrien KA, Sapir-Pichhadze R, Nakhla M, Ahmed SB, Dumanski SM, et al.. Hypertension Canada’s 2020 comprehensive guidelines for the prevention, diagnosis, risk assessment, and treatment of hypertension in adults and children.Can J Cardiol. 2020;36:596–624. 10.1016/j.cjca.2020.02.086. 10.1016/j.cjca.2020.02.086 32389335

[95] Umemura S, Arima H, Arima S, Asayama K, Dohi Y, Hirooka Y (2019). The Japanese Society of Hypertension Guidelines for the management of hypertension (JSH 2019. Hypertens Res.

[96] Raj M, Sundaram R, Paul M, Kumar K (2010). Blood pressure distribution in Indian children. Indian Pediatr.

[97] Sherif EM, El Maksood AAA, Youssef OI, Salah El-Din NY, Khater OKM (2019). Soluble urokinase plasminogen activator receptor in type 1 diabetic children, relation to vascular complications. J Diabetes Complications.

[98] Benmohammed K, Valensi P, Nguyen MT, Benmohammed F, Benlatreche M, Benembarek K, et al. Influence of waist circumference on blood pressure status in non-obese adolescents. Int J Adolesc Med Health. 2018;32(3).10.1515/ijamh-2017-0127. 10.1515/ijamh-2017-0127 29332014

[99] Katamba G, Agaba DC, Migisha R, Namaganda A, Namayanja R, Turyakira E (2020). Prevalence of hypertension in relation to anthropometric indices among secondary adolescents in Mbarara, Southwestern Uganda. Ital J Pediatr.

[100] Nsanya MK, Kavishe BB, Katende D, Mosha N, Hansen C, Nsubuga RN (2019). Prevalence of high blood pressure and associated factors among adolescents and young people in Tanzania and Uganda. J Clin Hypertens (Greenwich).

[101] Nyangasa MA, Buck C, Kelm S, Sheikh MA, Brackmann KL, Hebestreit A. Association between cardiometabolic risk factors and body mass index, waist circumferences and body fat in a Zanzibari cross-sectional study. BMJ Open. 2019;9(7):e025397. 10.1136/bmjopen-2018-025397. 10.1136/bmjopen-2018-025397 PMC6615808 31278089

[102] Leyvraz M, Wahlen R, Bloetzer C, Paradis G, Bovet P, Chiolero A (2018). Persistence of elevated blood pressure during childhood and adolescence: a school-based multiple cohorts study. J Hypertens.

[103] Muhihi AJ, Njelekela MA, Mpembeni RNM, Muhihi BG, Anaeli A, Chillo O (2018). Elevated blood pressure among primary school children in Dar es salaam, Tanzania: prevalence and risk factors. BMC Pediatr.

[104] Nakiriba R, Mayega RW, Piloya T, Nabukeera-Barungi N, Idro R (2018). Prevalence and factors associated with dysglycemia among girls in selected boarding secondary schools in Wakiso District. Uganda Adolesc Health Med Ther.

[105] Ndongala NJ, Maepa C, Nyondo E, Amstutz A, du Reau de la Gaignonnière B. Etiology, characteristics and occurrence of heart diseases in rural Lesotho (ECHO-Lesotho): A retrospective echocardiography cohort study. PLoS One. 2022;17(12):e0278406. 10.1371/journal.pone.0278406. 10.1371/journal.pone.0278406 PMC9754242 36520796

[106] Masocha V, Monyeki MA, Czyż SH. Longitudinal relationships between changes in body composition and changes in selected metabolic risk factors (abdominal obesity and blood pressure) among South African adolescents. Peer J. 2020;8:e9331. 10.7717/peerj.9331. 10.7717/peerj.9331 PMC7319020 32612883

[107] Jolliffe CJ, Janssen I (2007). Development of age-specific adolescent metabolic syndrome criteria that are linked to the Adult Treatment Panel III and International Diabetes Federation Criteria. JACC.

[108] Ostchega Y, Prineas RJ, Paulose-Ram R, Grim CM, Willard G, Collins D (2003). National Health and Nutrition Examination Survey 1999–2000: effect of observer training and protocol standardization on reducing blood pressure measurement error. J Clin Epidemiol.

[109] Arnaiz P, Müller I, Seelig H, Gerber M, Bosma J, Dolley D, et al. Practice change needed for the identification of pediatric hypertension in marginalized populations: an example from South Africa. Front Pediatr. 2022;10:877431. 10.3389/fped.2022.877431. 10.3389/fped.2022.877431 PMC9130957 35633959

[110] Köchli S, Smith W, Lona G, Goikoetxea-Sotelo G, Breet Y, Botha-Le Roux S (2022). Obesity, blood pressure and retinal microvascular phenotype in a bi-ethnic cohort of young children. Atherosclerosis.

[111] Ware LJ, Maposa I, Kolkenbeck-Ruh A, Norris SA, Soepnel L, Crouch S, et al. Are cardiovascular health measures heritable across three generations of families in Soweto, South Africa? A cross-sectional analysis using the random family method. BMJ Open. 2022;12(9):e059910. 10.1136/bmjopen-2021-059910. 10.1136/bmjopen-2021-059910 PMC9511591 36153021

[112] Chobanian AV, Bakris GL, Black HR, Cushman WC, Green LA, Izzo JL (2003). Seventh report of the Joint National Committee on Prevention, Detection, Evaluation, and Treatment of High Blood Pressure. Hypertension.

[113] Chungag A, Tata CM, Sewani-Rusike CR, Nel W, Nkeh-Chungag BN (2019). Ellisras Longitudinal Study 2017: association of hypertension with increasing levels of adiposity in 10- to 14-year-old boys and girls in the Eastern Cape (ELS 31). Cardiovasc J Afr.

[114] Mphekgwana PM, Monyeki KD, Makgopa HM, Makgae PJ (2020). Multiple points change in the association of blood pressure subtypes with anthropometric indices of adiposity among children in a rural population. Children (Basel).

[115] Nkwana MR, Monyeki KD, Monyeki SM, Makata TT, Monyeki JM (2019). Ellisras Longitudinal Study 2017: the association of fat patterning with blood pressure in Polokwane private school children aged five to 15 years (ELS 22). Cardiovasc J Afr.

[116] Houle B, Rochat TJ, Newell ML, Stein A, Bland RM. Breastfeeding, HIV exposure, childhood obesity, and prehypertension: a South African cohort study. PLoS Med. 2019;16(8):e1002889. 10.1371/journal.pmed.1002889. 10.1371/journal.pmed.1002889 PMC6711496 31454346

[117] Matjuda EN, Sewani-Rusike CR, Anye SNC, Engwa GA, Nkeh-Chungag BN (2020). Relationship between high blood pressure and microalbuminuria in children aged 6–9 years in a South African population. Children (Basel).

[118] Sebati B, Monyeki K, Makgae P (2020). An assessment of the relationship between anthropometric parameters and blood pressure among Polokwane private school children. Children (Basel).

[119] Bhimma R, Naicker E, Gounden V, Nandlal L, Connolly C, Hariparshad S (2018). Prevalence of primary hypertension and risk factors in grade XII learners in KwaZulu-Natal. South Africa Int J Hypertens.

[120] Meer R, Boateng D, Klipstein-Grobusch K, Norris SA, Kagura J (2022). Incidence and correlates of high blood pressure from childhood to adulthood: the Birth to Twenty study. J Hypertens.

[121] Gerber M, Müller I, Walter C, du Randt R, Adams L, Gall S (2018). Physical activity and dual disease burden among South African primary schoolchildren from disadvantaged neighbourhoods. Prev Med.

[122] Uchenwa-Onyenegecha TA, Gabriel-Job N (2021). Hypertension and pre-hypertension among children and adolescents in Port Harcourt. Nigeria West African Journal of Medicine.

[123] Azupogo F, Abizari AR, Aurino E, Gelli A, Osendarp SJM, Bras H (2020). Malnutrition, hypertension risk, and correlates: an analysis of the 2014 Ghana Demographic and Health Survey data for 15–19 years adolescent boys and girls. Nutrients.

[124] Abu OO, Raji YR, Amodu OK (2019). Risk factors for chronic kidney disease among in-school adolescents in Ibadan, Southwest. Nigeria Sahel Medical Journal.

[125] Musa DI, Toriola AL, Goon DT, Jonathan SU (2020). Association of fitness and fatness with clustered cardiovascular disease risk factors in nigerian adolescents. Int J Environ Res Public Health.

[126] Amponsem-Boateng C, Zhang W, Oppong TB, Opolot G, Kumi Duodu Kyere E. A cross-sectional study of risk factors and hypertension among adolescent Senior High School students. Diabetes Metab Syndr Obes. 2019;12:1173–80. 10.2147/DMSO.S213552. 10.2147/DMSO.S213552 PMC6662518 31413610

[127] Ibrahim OR, Afolabi JK, Adedoyin OT, Ojuawo AI (2019). Prevalence and risk factors for hypertension among school children in Ilorin. Northcentral Nigeria J Family Community Med.

[128] Abiodun O, Ladele A, Olu-Abiodun O, Ashipa T. Hypertension among adolescents in Nigeria: a retrospective study of adolescent university freshmen. Int J Adolesc Med Health. 2019;33(5). 10.1515/ijamh-2018-0287. 10.1515/ijamh-2018-0287 30875324

[129] Amadi OF, Okeke IB, Ndu IK, Ekwochi U, Nduagubam OC, Ezenwosu OU (2019). Hypertension in Children: Could the Prevalence be on the Increase?. Niger Med J.

[130] Adeomi AA, Adelusi IO, Adedeji PO, Awofeso AE, Oroleye OO, Gbadegesin DL (2019). Nutritional status and Cardiometabolic health among adolescents; findings from southwestern Nigeria. BMC Nutr.

[131] Schoenbuchner SM, Moore SE, Johnson W, Ngum M, Sonko B, Prentice A (2018). In rural Gambia, do adolescents have increased nutritional vulnerability compared with adults?. Ann N Y Acad Sci.

[132] Ezeudu CE, Chukwuka JO, Ebenebe JC, Igwe WC, Egbuonu I (2018). Hypertension and prehypertension among adolescents attending secondary schools in urban area of South-East. Nigeria Pan Afr Med J.

[133] Omisore AG, Omisore B, Abioye-Kuteyi EA, Bello IS, Olowookere SA (2018). In-school adolescents' weight status and blood pressure profile in South-western Nigeria: urban-rural comparison. BMC Obes.

[134] Iseuzo KO, Jiya NM, Audu Li, Ibitoye PK, Sani UM, Yusuf T, et al. Blood pressure pattern and the relationship with body mass index among apparently healthy secondary-school students in Sokoto metropolis, Nigeria. South African Journal of Child Health 2018; 12(3):1–6.

[135] Wariri O, Jalo I, Bode-Thomas F (2018). Discriminative ability of adiposity measures for elevated blood pressure among adolescents in a resource-constrained setting in northeast Nigeria: a cross-sectional analysis. BMC Obes.

[136] Chelo D, Mah EM, Chiabi EN, Chiabi A, Koki Ndombo PO, Kingue S (2019). Prevalence and factors associated with hypertension in primary school children, in the centre region of Cameroon. Transl Pediatr.

